# The *kpc-1* 3′UTR facilitates dendritic transport and translation efficiency of mRNAs for dendrite arborization of a mechanosensory neuron important for male courtship

**DOI:** 10.1371/journal.pgen.1011362

**Published:** 2024-08-07

**Authors:** Mushaine Shih, Yan Zou, Tarsis Ferreira, Nobuko Suzuki, Eunseo Kim, Chiou-Fen Chuang, Chieh Chang

**Affiliations:** 1 Department of Biological Sciences, University of Illinois at Chicago, Chicago, Illinois, United States of America; 2 School of Life Science and Technology, ShanghaiTech University, Shanghai, China; 3 Division of Developmental Biology, Cincinnati Children’s Hospital Research Foundation, Cincinnati, Ohio, United States of America; University of California San Diego, UNITED STATES OF AMERICA

## Abstract

A recently reported Schizophrenia-associated genetic variant in the 3′UTR of the human furin gene, a homolog of *C*. *elegans kpc-1*, highlights an important role of the furin 3′UTR in neuronal development. We isolate three *kpc-1* mutants that display abnormal dendrite arborization in PVD neurons and defective male mating behaviors. We show that the *kpc-1* 3′UTR participates in dendrite branching and self-avoidance. The *kpc-1* 3′UTR facilitates mRNA localization to branching points and contact points between sibling dendrites and promotes translation efficiency. A predicted secondary structural motif in the *kpc-1* 3′UTR is required for dendrite self-avoidance. Animals with over-expression of DMA-1, a PVD dendrite receptor, exhibit similar dendrite branching and self-avoidance defects that are suppressed with *kpc-1* over-expression. Our results support a model in which KPC-1 proteins are synthesized at branching points and contact points to locally down-regulate DMA-1 receptors to promote dendrite branching and self-avoidance of a mechanosensory neuron important for male courtship.

## Author summary

Here, we report a non-coding sequence immediately downstream of the *kpc-1* gene participating in the transport and, subsequently, the translation of the *kpc-1* messenger RNA in the nerve process of a mechanosensory neuron, which is required to establish a functional neuronal circuit regulating the male mating behavior in *C*. *elegans*. Interestingly, a human Schizophrenia-associated mutation located in the non-coding sequence immediately downstream of the human furin gene, a *kpc-1* homologous gene, was recently shown to contribute to the pathogenesis of Schizophrenia. By analogy, whether this human furin downstream mutation disrupts the transport and the translation of the furin mRNA in the nerve process of Schizophrenia-causing neurons during its differentiation remains to be seen.

## Introduction

PVD nociceptive neurons innervate *C*. *elegans* skin with elaborate dendrite arbors that respond to harsh mechanical stimuli [1]. Dendrite arborization, the intricate process of dendrite branching during neuronal development, allows uniform sensory coverage of the skin by PVD neurons. This developmental process ensures that the dendrites of PVD sensory neurons envelop the entire surface of the animal body outside the head region. However, the mechanism underlying this process is still largely unknown. Dendrite arborization in PVD neurons is restricted within a defined time frame by two intrinsic timing mechanisms [2] and is constrained spatially by two organizing principles: Escape (branch out) from the intermediate target (the trap zone) upon arrival at it and self-avoidance [3–5]. Self-avoidance is a process in which their repulsion from sibling branches follows self-recognition among neurites of a single neuron. This selective repulsion between dendrites of the same cell was originally observed in leech neurons and proposed to promote a uniform receptive field [6,7]. Recent studies using *Drosophila* as a model showed that the homophilic binding of Dscam1 proteins on sibling branches of the same cell (MB or da neurons) promotes repulsive interactions between them to ensure that they diverge and grow along separate pathways [8–12]. Since the Dscam1 mutation does not completely disrupt dendrite self-avoidance in *Drosophila* and there is no Dscam1 homolog in *C*. *elegans*, studying PVD dendrite self-avoidance in *C*. *elegans* provides a unique opportunity to identify novel mechanisms of dendrite self-avoidance. Indeed, several known developmental pathways, including UNC-6 (netrin), MIG-14 (Wntless), and FMI-1 (Flamingo) signaling, have been implicated in PVD dendrite self-avoidance in *C*. *elegans* [5,13,14].

**Fig 1:**
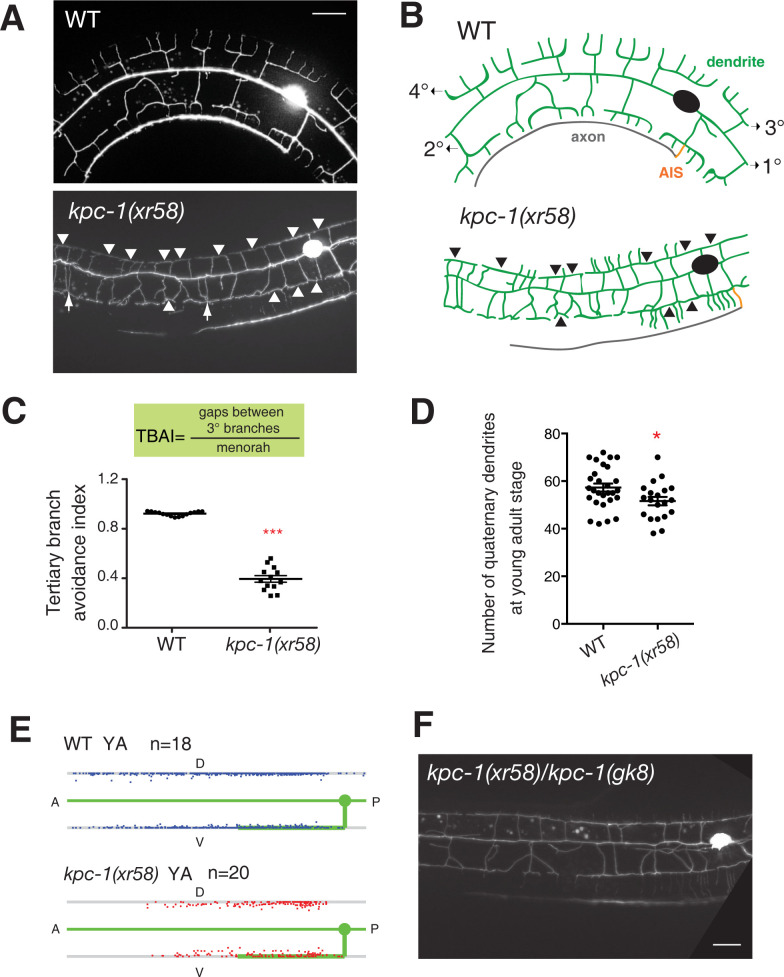
*kpc-1(xr58)* mutations mainly affect PVD dendrite self-avoidance. (A) Images of PVD dendrites in wild-type and *kpc-1(xr58)* mutants. Scale bar, 20 μm. Arrows point to contacts between neighboring secondary dendrites. Arrowheads point to contacts between neighboring tertiary dendrites. (B) Schematic of PVD dendrites (green), axon (grey), and axon initial segment (AIS; orange). PVD projected primary (1°) dendritic processes anteriorly and posteriorly from the soma. Higher-order dendritic branching (secondary 2°, tertiary 3°, quaternary 4°) alternated between anterior-posterior and dorsal-ventral directions to generate a regularly spaced array of parallel branches. *kpc-1(xr58)* mutants, while showing effective secondary dendrite branching, displayed extensive defects in the self-avoidance of sibling dendrites. (C) Tertiary branch avoidance index (TBAI) in wild-type versus *kpc-1(xr58)* mutants. *kpc-1(xr58)* mutants displayed significantly lower TBAI than wild-type animals. ***p < 0.001 by a Student’s *t*-test. Error bars indicate SEM. (D) Number of quaternary dendrites measured anterior to the cell body in wild-type and *kpc-1(xr58)* mutants. *p < 0.05 by a Student’s *t*-test. Error bars indicate SEM. (E) Scatter plots showing the positions of 4° dendrite termini in wild-type young adults and *kpc-1(xr58)* young adults. In each scatter plot, the top line (dorsal nerve cord), the bottom line (ventral nerve cord), and the wild-type PVD axon and primary dendrite (green) are shown. (F) Image of the trans-heterozygous *kpc-1(xr58)/kpc-1(gk8)* mutant displaying self-avoidance defects similar to those displayed by homozygous *kpc-1(xr58)* mutants. Scale bars, 20 μm.

Guidance cues SAX-7 (L1CAM) and MNR-1 (Menorin) generated in the hypodermis (skin) established dendrite growth pathways and instructed dendrite arborization for PVD neurons in *C*. *elegans* [15,16]. PVD neurons first grow two longitudinally extending 1° dendrites along the anterior-posterior (A/P) axis. Orthogonal arrays of 2°, 3°, and 4° dendritic branches emerge sequentially in a manner that alternates between the dorsal-ventral (D/V) and the A/P axes to produce an elaborate network of sensory processes [4,17] (Fig 1B). Skin cues, SAX-7 and MNR-1, and a muscle-derived cue, LECT-2, are required for dendrite pattern formation through direct interactions with the dendrite receptor DMA-1 to form a multi-protein receptor-ligand complex [15,16,18–20].

Many studies have shown that specific elements in 5′ or 3′ untranslated regions (UTRs) of mRNA regulate mRNA stability, splicing, translation and localization [21]. microRNAs regulate mRNA translation and stability by binding to complementary sequences in the 3′UTR [22]. Other types of elements in the 3′UTR can function in either a sequence- or secondary structure-specific manner. For example, one of the earliest identified localization elements was discovered from the chick β-actin mRNA. The “zipcode” in the 3′ UTR of β-actin mRNA consists of 54 nucleotides that contain tandem repeats of the conserved hexanucleotide motif ACACCC and form a stem-loop structure recognized by the Zipcode Binding Proteins (ZBPs) to regulate the localization and translation of β-actin mRNA [23,24]. In yeast, the cis-acting sequences that mediate ASH1 mRNA targeting to the bud tip provide another example of repetitive clustering and synergistic action of localization elements. Four localization elements in ASH1 mRNA were all predicted to form stem-loop structures, and each element on its own is able to localize mRNAs, but the presence of four elements together increases the efficiency of localization [25–27]. In Aplysia sensory-motor neurons, cis-elements in the 3′UTR of the neurotransmitter sensorin mRNA control the transport of sensorin mRNAs from soma to neurite [28]. In mice, conditional deletion of an axon-localizing cis-element in the 3′UTR of importin β1 mRNAs in sensory neurons caused selective depletion of importin β1 mRNAs and proteins in axons, without affecting cell body levels or nuclear functions, suggesting that local translation of importin β1 mRNAs in axons enables separation of axonal and nuclear transport functions of importins, and is required for efficient retrograde signaling in the injured axon [29].

In *C*. *elegans*, the *cebp-1* 3′UTR is required for the localization of *cebp-1* mRNAs to discrete locations in touch axons, which can be translated into proteins locally [30]. What is unclear is whether those discrete locations represent synapses in touch axons. In addition, a putative stem-loop secondary structure was identified in the *cebp-1* 3′UTR that inhibits *cebp-1* mRNA stability or its translation [31]. Thus, the *cebp-1* 3′UTR appears to have both positive and negative impacts on *cebp-1* gene expression. In this study, we characterized the role of the *kpc-1* 3′UTR in the context of dendrite branching and self-avoidance.

We isolate mutants through genetic screens that display profoundly affected dendrite branching and self-avoidance in PVD neurons. We identify mutations in the *kpc-1* gene as being responsible for defective dendrite branching and self-avoidance phenotypes through whole genome sequencing. *kpc-1* mutants display a significantly lower tertiary branch avoidance index and a decreased sensory coverage in the skin compared to wild-type animals. Although *kpc-1* has been implicated in dendrite self-avoidance and arborization [3,32,33], the spatial mechanism by which *kpc-1* regulates these processes is poorly understood. KPC-1 encodes a proprotein convertase subtilisin/kexin (PCSK). Here, we show that *kpc-1* functions in PVD neurons to regulate dendrite branching and self-avoidance via the *kpc-1* 3′UTR. The *kpc-1* 3′UTR is required for *kpc-1* mRNA localization to distal and higher-order dendrites and promotes its local translation efficiency. Although over-expression of *kpc-1* causes greater self-avoidance, it does not limit initial dendrite outgrowth, which supports a direct role for *kpc-1* in self-avoidance. *dma-1* over-expression also displays similar secondary dendrite branching and tertiary dendrite self-avoidance defects that are suppressed with *kpc-1* over-expression. Thus, DMA-1 is a potential KPC-1 target that becomes down-regulated with increased KPC-1 activity. Our results suggest a model in which KPC-1 proteins are synthesized at branching points and contact points between sibling dendrites, leading to local down-regulation of DMA-1 receptors and the consequent dendrite branching and self-avoidance. At a functional level, studies in *kpc-1* mutant males revealed that *kpc-1* patterns PVD neurons to coordinate diverse behavioral motifs in reproductive behavior and that male mating behavior requires mechanosensory inputs from PVD neurons to generate proper mating motor patterns.

## Results

### Isolation of mutants that display profound self-avoidance defects in dendrites of PVD neurons

*C*. *elegans* PVD neurons extend a large and highly branched dendrite arbor directly beneath the hypodermal surface that envelops the worms to mediate an avoidance response to harsh mechanical force as well as to other noxious stimuli including temperature [1]. The elaborate dendrite branching patterns that PVD neurons display are constrained by two organizing principles: dendrite branching [3] and dendrite self-avoidance. Self-avoidance is an important neuronal process in which dendritic branches arising from a single soma (also called isoneuronal) turn away from one another to minimize crossing and overlap and to promote a uniform receptive field [34].

The self-avoidance in *C*. *elegans* is likely to provide new mechanistic insight into the process as the well-known self-avoidance molecule Dscam [8–12]is absent from the *C*. *elegans* genome. To uncover new mechanisms responsible for self-avoidance in *C*. *elegans*, we screened approximately 9,500 genomes for mutants that displayed dendrite self-avoidance defects. In our genetic screens, we focused on identifying strong mutants with profound self-avoidance defects in secondary and tertiary dendrites and recovered only three such mutants (*xr47*, *xr58*, and *xr60*). All of them displayed 100% penetrance of self-avoidance defects (n>200 examined each; S1 Fig). *xr47* and *xr60* mutants showed an additional secondary dendrite branching phenotype (a defect in the dendrite branching), which resulted in a significantly reduced number of secondary branches in *xr47* and *xr60* mutants compared to wild-type animals (S1A, S1C and S1D Fig). Despite displaying extensive defects in self-avoidance among secondary and tertiary sibling dendrites, *xr58* mutants showed little secondary dendrite branching defect (Figs 1A, S1H and S1I). In wild-type animals, secondary dendrites that initially contacted neighboring secondary branches retracted to form a regularly spaced array of parallel branches (Fig 1A and 1B). In *xr58* mutants, many secondary dendrites remained in contact with each other in the adult stage (Fig 1A). In wild-type animals, tertiary dendrites are initially extended but later retracted once their termini come into contact with sibling tertiary branches (S2 Fig). In *xr58* mutants, many tertiary dendrites remained in contact with their siblings long after arborization ended (Figs 1A and S2), resulting in a significantly lower tertiary branch avoidance index (TBAI) compared to the wild-type animals (Fig 1C; TBAI: 0.38 in *xr58* mutants versus 0.93 in wild-type). Moreover, we observed a moderate reduction of quaternary dendrites in *xr58* mutants, which can be partly attributed to the undercounted quaternary dendrites that grew up from the overlapped tertiary dendrite area in the *kpc-1(xr58)* mutant allele (Fig 1D). In wild-type animals, PVD dendrite arbors were fully extended beneath the hypodermis and covered the areas from the posterior animal body to the area right before the head region (Figs 1E and S3A). In *xr58* mutants, PVD dendrite arbors developed partially overlapping menorahs, causing decreased sensory coverage of skin (Fig 1E), judged by less skin area covered by PVD sensory dendritic arbors (S3B Fig). We quantified sensory coverage of skin by PVD dendrite arbors using receptive field index, and the measurement showed significantly reduced values in *kpc-1(xr58)* mutants compared to wild-type animals (S3C Fig). A summary of the *xr58* mutant’s phenotype is shown in S3D Fig.

### Identification of responsible mutations in the *kpc-1* gene

**Fig 2:**
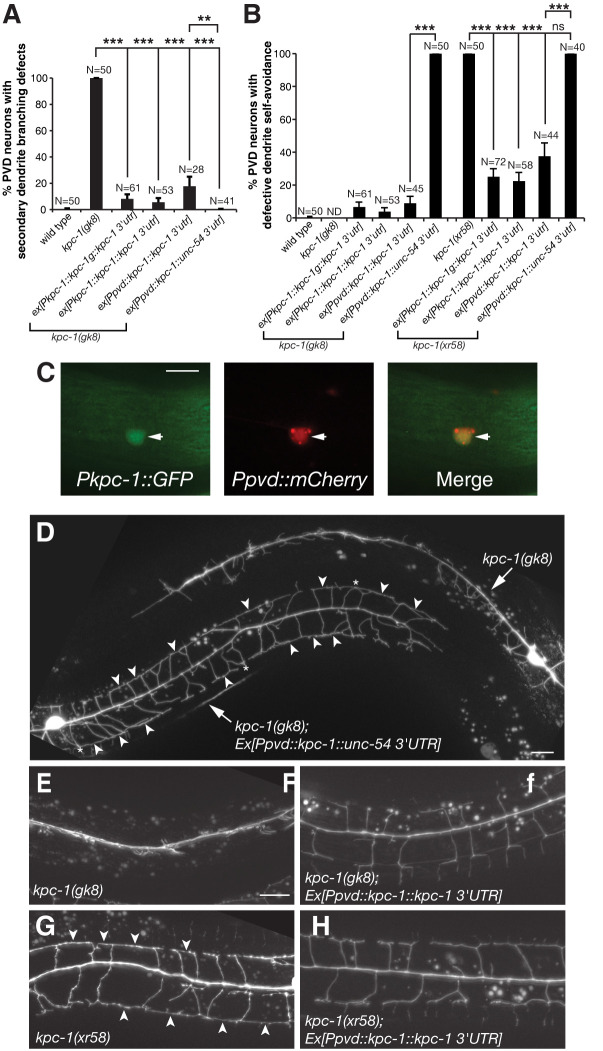
*kpc-1* is required for both secondary dendrite branching and tertiary dendrite self-avoidance. (A) *kpc-1* functions in PVD neurons to promote secondary dendrite branching largely independent of the *kpc-1* 3′UTR. Percentage PVD neurons with secondary dendrite branching defects in *kpc-1(gk8)* mutants carrying various transgenes. **p < 0.01 and ***p < 0.001 by a two-proportion *Z*-test. (B) The *kpc-1*′s function in PVD for tertiary dendrite self-avoidance depends on the *kpc-1* 3′UTR. Percentage PVD neurons with defective tertiary dendrite self-avoidance in *kpc-1(gk8)* and *kpc-1(xr58)* mutants carrying various transgenes. ND, not determined. ***p < 0.001 by a two-proportion *Z*-test. (C) Identification of the *kpc-1* promoter-reporter expression in PVD neurons. (D) Images of PVD neurons in *kpc-1(gk8)* mutants with or without the *Ppvd*::*kpc-1*::*unc-54* 3′UTR transgene. The *Ppvd*::*kpc-1*::*unc-54* 3′UTR transgene rescued the secondary dendrite branching but not the tertiary dendrite self-avoidance defect in *kpc-1(gk8)* mutants. Arrowheads point to contacts between neighboring tertiary dendrites. Asterisks indicate contacts between neighboring secondary dendrites. (E-F) Images of PVD neurons in *kpc-1(gk8)* mutants without or with the *Ppvd*::*kpc-1*::*kpc-1* 3′UTR transgene. The *Ppvd*::*kpc-1*::*kpc-1* 3′UTR transgene rescued both the secondary dendrite branching and the tertiary dendrite self-avoidance defect in *kpc-1(gk8)* mutants. (G-H) Images of PVD neurons in *kpc-1(xr58)* mutants without or with the *Ppvd*::*kpc-1*::*kpc-1* 3′UTR transgene. The *Ppvd*::*kpc-1*::*kpc-1* 3′UTR transgene rescued the tertiary dendrite self-avoidance defect in *kpc-1(xr58)* mutants. Arrowheads point to contacts between neighboring tertiary dendrites. Scale bar, 20 μm.

We identified *xr47*, *xr58*, and *xr60* mutations in the *kpc-1* gene through whole genome sequencing. The *xr58* allele harbors a missense mutation resulting in a P440S amino acid substitution at a conserved proline residue four amino acids away from the catalytic serine residue toward the C-terminal [3]. *kpc-1(my24)*, a different mutation affecting the same residue (P440L), caused a loss of function in the *kpc-1* gene, which resulted in defective secondary dendrite branching in PVD neurons [33]. Thus, it appears that mutations at P440 could alter KPC-1 functions moderately or severely. Using the complementation test, we showed that the *kpc-1(gk8)* null mutation failed to complement the *xr58* mutant phenotype in PVD neurons, further confirming that *xr58* is a new *kpc-1* allele (Fig 1F). The *xr58* mutant reveals an additional function of *kpc-1* in tertiary dendrite self-avoidance as trans-heterozygous mutants (*xr58/gk8*) display the *xr58* mutant phenotype of defective tertiary dendrite self-avoidance rather than defective secondary dendrite branching characteristic of *gk8* (Figs 1A and 2D). The two other *kpc-1* alleles are also predicted to affect KPC-1 protein structure and function. The *xr60* allele contains a missense mutation, which results in a G223S amino acid substitution in the catalytic domain, whereas the *xr47* allele contains a G-to-A transition at the +1 position of intron 5, which abolishes the donor splice site (S1F Fig). Compared to the *gk8* null allele, *xr58*, *xr47*, and *xr60* alleles are weaker in their phenotypic effects in PVD neurons as judged by the extent of menorah coverage and trapped secondary dendrites along the primary dendrite axis (S1 Fig).

### Elements in the *kpc-1* 3′UTR are required for dendrite self-avoidance

The *kpc-1* genomic DNA containing the 5′, coding, and 3′ fragments rescued both defective secondary dendrite branching and defective tertiary dendrite self-avoidance in the *kpc-1(gk8)* null allele as well as defective tertiary dendrite self-avoidance in the *kpc-1(xr58)* allele (Fig 2A and 2B). To investigate the genomic requirement of the *kpc-1* gene in different steps of PVD dendrite development, we first reduced the *kpc-1* gene to the *kpc-1* coding region without introns. We found that the *kpc-1* gene without introns (cDNA) led to a complete rescue of the dendrite phenotypes in both *kpc-1(gk8)* null and *kpc-1(xr58)* alleles (Fig 2A and 2B). We then further replaced the *kpc-1* promoter with a PVD cell-specific promoter (The F49H12.4 promoter). This *Ppvd*::*kpc-1*::*kpc-1 3*′*UTR* transgene remained able to cause a near complete phenotypic rescue, in a manner similar to the *kpc-1* genomic DNA, in both *kpc-1(gk8)* null and *kpc-1(xr58)* alleles, indicating that *kpc-1* acts cell-autonomously in PVD (Fig 2A, 2B, and 2E–2H). Consistent with a cell-autonomous role of *kpc-1* in PVD, the expression of the *kpc-1* promoter reporter was detected in PVD neurons during secondary dendrite branching and tertiary dendrite self-avoidance (Fig 2C).

When the *kpc-1* 3′UTR was replaced with an unrelated *unc-54* 3′UTR, the *Ppvd*::*kpc-1*::*unc-54* 3′UTR transgene was able to largely, albeit incompletely, rescue defective secondary dendrite branching in the *kpc-1(gk8)* null allele (Fig 2A, 2B and 2D). However, the *Ppvd*::*kpc-1*::*kpc-1* 3′UTR transgene rescued better than the *Ppvd*::*kpc-1*::*unc-54* 3′UTR transgene the *kpc-1(gk8)* mutant phenotype of dendrite self-avoidance defects (Fig 2A and 2B), suggesting that the *kpc-1* 3′UTR is required for tertiary dendrite self-avoidance. In addition to tertiary dendrites, some secondary dendrites displayed self-avoidance defects in these transgenic animals (Fig 2D). The same *Ppvd*::*kpc-1*::*unc-54 3*′*UTR* transgene was also unable to rescue the defective tertiary dendrite self-avoidance in the *kpc-1(xr58)* allele (Fig 2B). The *unc-54* 3′UTR was chosen as a control since no apparent conserved stem-loop structure was identified in the *unc-54* 3′UTR based on an analysis using Turbofold II-predicted secondary structures [35] and sequence alignment of the *unc-54* 3′UTRs from 4 closely related nematode species (*C*. *elegans*, *C*. *remanei*, *C*. *briggase*, and *C*. *japonica*; S4 Fig). We also used the 3′UTR of DMA-1, a dendrite receptor gene, as additional control and found that the *Ppvd*::*kpc-1*::*dma-1* 3′UTR transgene, like the *Ppvd*::*kpc-1*::*unc-54 3*′*UTR* transgene, failed to rescue the tertiary dendrite self-avoidance defect in the *kpc-1(xr58)* mutants (S5 Fig). Thus, these results indicate that tertiary dendrite self-avoidance depends on elements in the *kpc-1* 3′UTR.

### The *kpc-1* 3′UTR is required for localization of *kpc-1* mRNAs to distal primary dendrites and higher-order tertiary dendrites in PVD neurons

**Fig 3:**
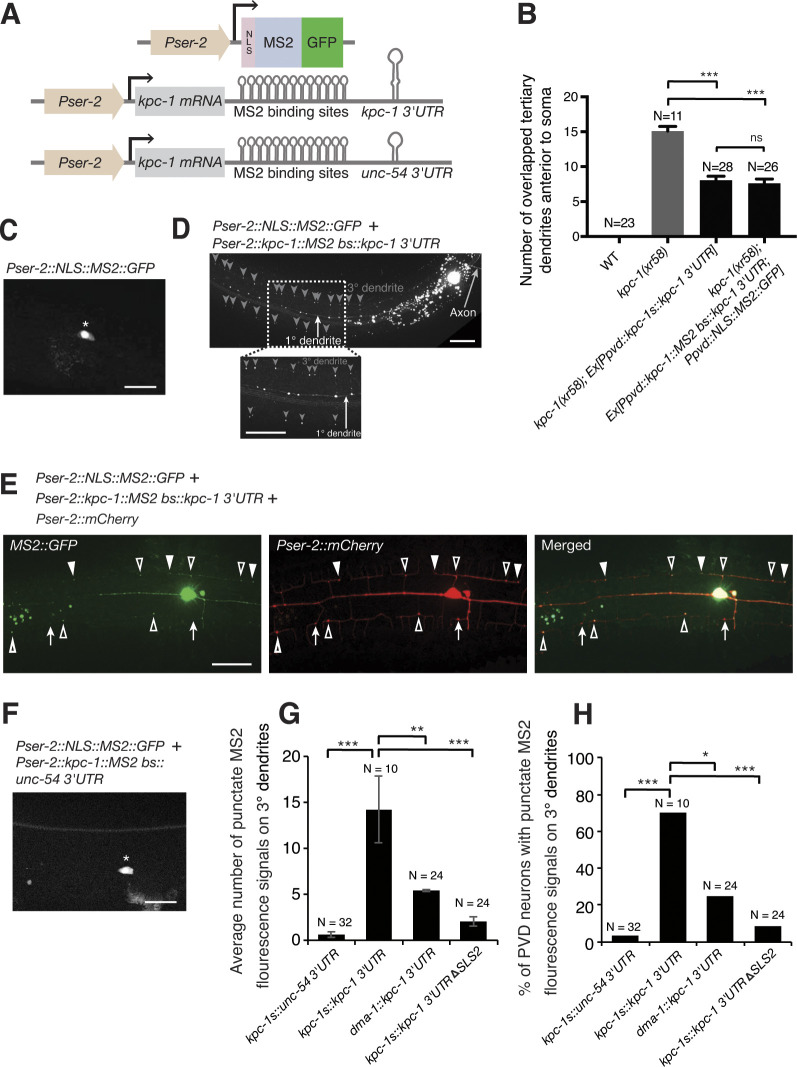
The *kpc-1* 3′UTR targets the *kpc-1* mRNA to higher-order dendrites. (A) The MS2 tagging system comprises the *kpc-1* mRNA containing its own 3′UTR or a control *unc-54* 3′UTR fused to 24xMS2 binding sites co-expressed with a green fluorescent MS2 capsid protein. The nuclear localization signal (NLS) is used to initially target green fluorescent MS2 proteins to the nucleus. (B) Quantification of overlapped tertiary dendrites 200 μm anterior to soma in wild-type and *kpc-1(xr58)* mutants carrying various versions of transgenes. ***p<0.001 by one-way ANOVA with Dunnett’s test. The N number indicates the number of animals analyzed. (C) Image of the PVD neuron showing enriched nuclear signals for the nuclear-localized green fluorescent MS2 capsid protein. Asterisk indicates soma. Scale bar, 20 μm. (D) Image of the PVD neuron showing axonal and dendritic signals for *kpc-1* transcripts containing the *kpc-1* 3′UTR and MS2 binding sites. The white arrow points to dendritic signals in the primary dendrite, and the grey arrow marks axonal signals. Grey arrowheads indicate the potential branching points at 3° dendrites and contact points between neighboring 3° dendrites. Anterior is to the left, dorsal is up. Scale bar, 20 μm. (E) Image of the PVD neuron showing axonal and dendritic signals for *kpc-1* transcripts containing the *kpc-1* 3′UTR and MS2 binding sites. Low level of the mCherry protein was used to label PVD. Arrowheads point to potential tertiary dendrite contact points, and open arrowheads denote dendrite branching points. Arrows show red dendrite markers that are not overlapped with any green MS2::GFP signals, indicating that red signals did not bleed through to give rise to green signals. Scale bar, 20 μm. (F) Image of the PVD neuron showing enriched soma signals for *kpc-1* transcripts containing the control *unc-54* 3′UTR and MS2 binding sites. Asterisk indicates soma. Scale bar, 20 μm. (G) The average number of punctate MS2 fluorescence signals on 3° dendrites in transgenic animals expressing transcripts containing MS2 binding sites with different coding genes and 3′UTRs. **p < 0.01 and ***p < 0.001 by a Student’s *t*-test. Error bars indicate SEM. (H) Percentage of PVD neurons with punctate MS2 fluorescence signals on 3° dendrites in transgenic animals expressing transcripts containing MS2 binding sites with different coding genes and 3′UTRs. *p < 0.05 and ***p < 0.001 by a two-proportion *Z*-test.

To directly visualize *kpc-1* mRNA distribution in PVD neurons, we utilized the MS2 tagging system, which relies on the sequence-specific interaction between the MS2 bacteriophage RNA hairpin loops and capsid proteins. In this system, the *kpc-1* mRNA containing either its own 3′UTR or a control *unc-54* 3′UTR was fused to tandem MS2 binding sites and co-expressed with a green fluorescent MS2 capsid fusion protein [36,37] (Fig 3A). We showed that the *kpc-1* mRNA containing tandem MS2 binding sites rescued the self-avoidance defect in the *kpc-1(xr58)* mutants to a similar extent to the *kpc-1* mRNA that does not contain tandem MS2 binding sites, suggesting that using the MS2 tagging system to visualize the *kpc-1* mRNA distribution is physiologically relevant (Fig 3B). In this system, the green fluorescent MS2 capsid fusion protein, which is targeted to the nucleus by the nuclear localization signal (Fig 3C), binds tightly to MS2 binding sites fused to the 3′UTR of the *kpc-1* mRNA, allowing visualization of the *kpc-1* mRNA by MS2 fluorescence in PVD neurons. We detected significant axonal and dendritic signals for *kpc-1* transcripts containing its own 3′UTR (Fig 3D and 3G). Dendritic signals were preferentially distributed in the 1° and 3° dendrites (Fig 3D), consistent with *kpc-1*’s roles in regulating secondary dendrite branching from the 1° dendrites and tertiary dendrite self-avoidance. The distribution of *kpc-1* mRNAs in 3° dendrites was region-specific. We observed enriched localization at potential branching points and retracted contact points between neighboring 3° dendrites (Fig 3D). Using the mCherry reporter to label PVD dendrites, we can confirm enrichment of *kpc-1* transcripts at branching points and retracted contact points (Fig 3E). In contrast, when the *kpc-1* 3′UTR was replaced with an unrelated *unc-54* 3′UTR, the *kpc-1* transcripts were mainly trapped in the nucleus, with some perinuclear signals detected in PVD (Fig 3F). *kpc-1* mRNAs containing the *unc-54* 3′UTR had a significantly lower chance of being transported to higher-order tertiary dendrites compared to *kpc-1* mRNAs containing the *kpc-1* 3′UTR (Fig 3G and 3H). To determine whether the *kpc-1* 3′UTR is sufficient to localize RNA to higher-order dendrites, we analyzed the expression of the *Pser-2*::*dma-1*::*ms2*::*kpc-1 3*′*UTR* transgene, in which the *kpc-1* coding region was replaced with the coding region of the *dma-1* dendrite receptor gene. We found that this transgene, while retaining partial localization of the *dma-1* mRNA to the higher-order dendrite, reduced the extent of dendritic localization (Fig 3G and 3H), suggesting that the *kpc-1* 3′UTR alone is not sufficient to fully localize RNAs to higher-order dendrites. Together, these results indicate that both the *kpc-1* 3′UTR and the *kpc-1* coding region facilitate the dendritic transport of *kpc-1* mRNAs. Consistent with this interpretation, both the *kpc-1* 3′UTR deletion and a mutation in the *kpc-1* coding region, such as the *kpc-1(xr58)* mutation located in the catalytic domain, result in dendrite self-avoidance defects. Since *kpc-1* mRNAs are localized to distal primary dendrites and higher-order tertiary dendrites, our results implicate the local translation of *kpc-1* in dendrites.

### The *kpc-1* 3′UTR promotes mRNA translation efficiency in the distal segment of PVD dendrites

**Fig 4:**
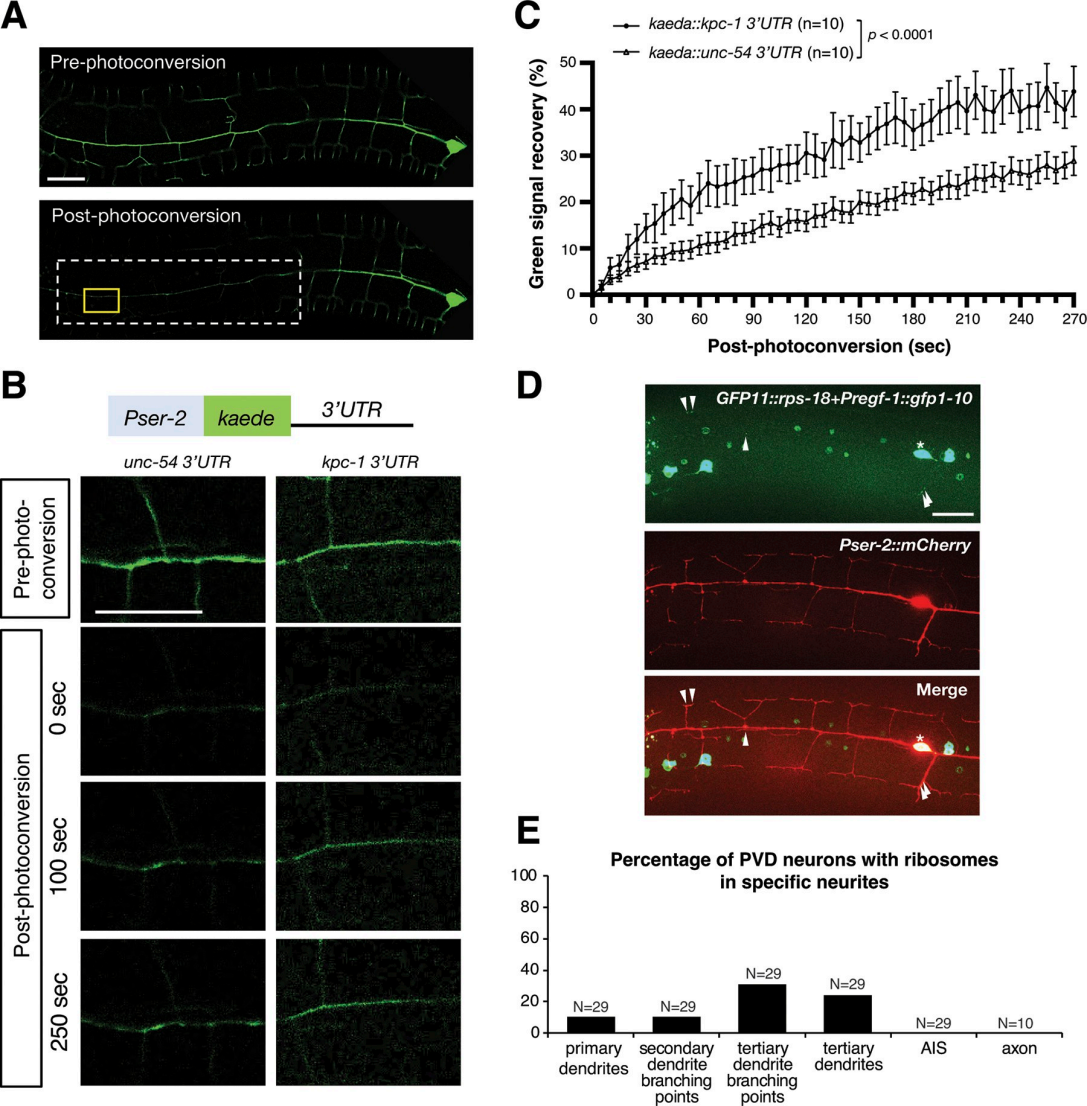
The *kpc-1* 3′UTR promotes local mRNA translation efficiency in the distal segment of PVD dendrites. (A) Representative images of Kaeda-labelled PVD dendrites pre- and post-photoconversion. The dashed white box indicates a wide photoconverted region. The solid yellow box denotes a targeted area of analysis, which is distal to the soma within the wide photoconverted region. Scale bar, 20 μm. (B) Pre-photoconversion of resident Kaede proteins and post-photoconversion of newly synthesized Kaede proteins. Kaeda expression in PVD dendrites is controlled by either a control *unc-54* 3′UTR or a *kpc-1* 3′UTR. Scale bar, 20 μm. (C) The *kaeda*::*kpc-1 3*′*UTR* transcript is expressed at a higher rate compared to the control *kaeda*::*unc-54 3*′*UTR* transcript. Quantification of green signal recovery in a targeted distal area after photoconversion of a wider region of PVD dendrites. Statistical analysis using two-way ANOVA. Average data of Kaeda expression intensity are presented as means ± SEM. (D) Expression of a split GFP reporter allows visualization of ribosomes in PVD neurons. The *Pser-2*::*mCherry* marker labels PVD dendrites. Ribosomes are mostly restricted in the PVD soma, which is marked by an asterisk. Arrowheads indicate RPS-18 signals. Scale bar, 20 μm. (E) Percentage of PVD neurons with ribosomes in specific neurites. Animals were analyzed at L4 to young adult stage.

Our results showed that the *kpc-1* 3′UTR facilitates localization of *kpc-1* mRNAs to the distal primary and tertiary PVD dendrites, suggesting that the *kpc-1* 3′UTR promotes local translation of transcripts it regulates. To examine whether the *kpc-1* 3′UTR can promote local protein synthesis, we performed fluorescence recovery after photoconversion (FRAP) of the photoconvertible Kaede protein [38], which was under the control of the *kpc-1* 3′UTR or a control *unc-54* 3′UTR. Upon photostimulation using 405 nm laser, Kaede proteins can be converted from green fluorescent proteins to red fluorescent proteins irreversibly, allowing us to distinguish the future newly synthesized green fluorescent Kaede proteins from the resident (converted) red fluorescent Kaede proteins. We carried out photoconversion at a broad region from the mid-body to the nerve ring and measured newly synthesized green fluorescent Kaede proteins in a local area distant away from the cell body (Fig 4A). This design ensures that the local green fluorescence signal represents newly synthesized Kaede proteins rather than resident (unconverted) Kaede proteins diffused from the soma (Fig 4A). Green fluorescence recovery of Kaede proteins expressed from the *kaede*::*kpc-1* 3′UTR is significantly faster than that from the *kaede*::*unc-54* 3′UTR (Fig 4B and 4C), indicating that the *kpc-1* 3′UTR promotes local protein translation efficiency in the distal segment of PVD dendrites. Using the split GFP *rps-18* reporter to visualize ribosomes in PVD neurons [39], we detected the protein translation machinery not only in the soma but also in primary dendrites, secondary dendrites, tertiary dendrites (Fig 4D and 4E), further supporting protein synthesis occurring in the higher order dendrites to allow for proteome remodeling. We did not detect the split GFP *rps-18* signal in axons or axon initial segments (Fig 4E), suggesting that local protein synthesis mainly occurs in dendrites rather than axons in PVD neurons.

### Identification and characterization of secondary structural motifs in the *kpc-1* 3′UTR required for tertiary dendrite self-avoidance

**Fig 5:**
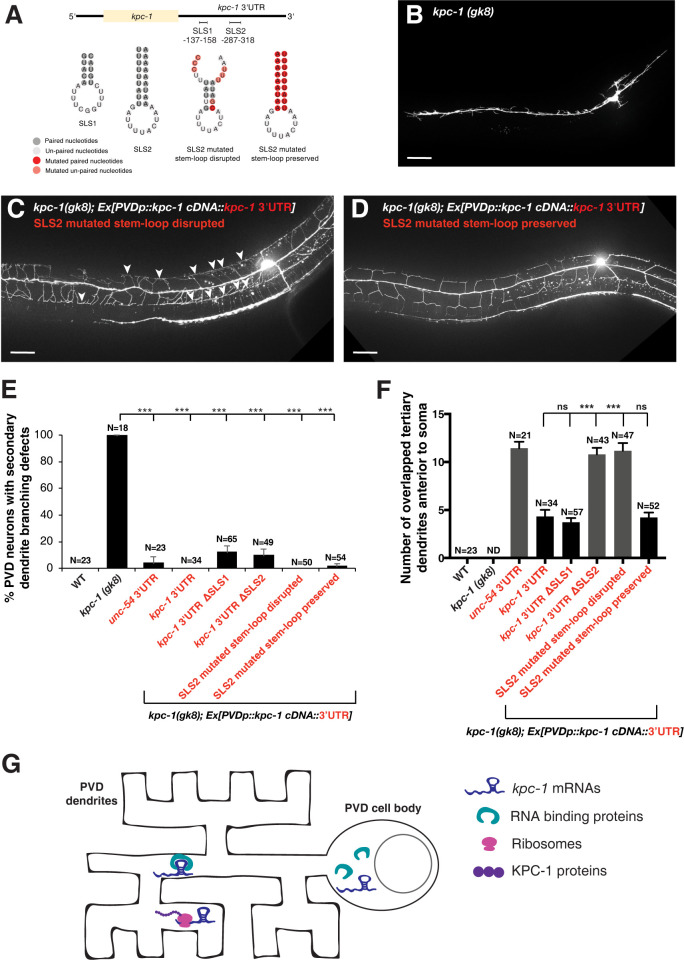
A putative stem-loop secondary structure in the *kpc-1* 3′UTR is required for dendrite self-avoidance. (A) Illustration of putative stem-loop secondary structural motifs and specific mutations in the *kpc-1* 3′UTR. (B-D) Representative images of the *kpc-1(gk8)* mutant, the *kpc-1(gk8)* mutant expressing the *Ppvd*::*kpc-1*::*kpc-1* 3′UTR transgene (SLS2 mutated base pairing disrupted), and the *kpc-1(gk8)* mutant expressing the *Ppvd*::*kpc-1*::*kpc-1* 3′UTR transgene (SLS2 mutated base pairing preserved). Arrowheads point to contacts between neighboring tertiary dendrites. Scale bars, 20 μm. (E) Percentage PVD neurons with secondary dendrite branching defects in wild-type, *kpc-1(gk8)* mutants, and *kpc-1(gk8)* mutants carrying various transgenes. ***p < 0.001 by a two-proportion *Z*-test. The N number indicates the number of animals analyzed. (F) Quantification of overlapped tertiary dendrites 200 μm anterior to soma in wild-type and *kpc-1(gk8)* mutants carrying various transgenes. ND, not determined. ***p<0.001 by one-way ANOVA with Dunnett’s test. The N number indicates the number of animals analyzed. (G) The schematic drawing illustrating the *kpc-1* mRNA containing a stem-loop secondary structure in its 3′UTR can be transported to higher-order dendrites through an unknown RNA binding protein and subsequently be translated locally.

Secondary structural motifs derived from the alignment of *kpc-1* 3′UTRs from 4 closely related nematode species, including *C*. *elegans* (S4A Fig), as well as individual Turbofold II-predicted secondary structures [35], revealed that two stem-loop structural motifs (SLS1 and SLS2) are conserved in the 3′UTR of all *kpc-1* nematode homologs (Fig 5A). The *kpc-1* transgene with its 3′UTR being replaced by a control *unc-54* 3′UTR lost the ability to rescue tertiary dendrite self-avoidance defects in the *kpc-1(gk8)* null allele (Fig 5F). Deletion analysis showed that SLS2 but not SLS1 is required for the *kpc-1* 3′UTR to mediate tertiary dendrite self-avoidance since Δ*SLS2* but not Δ*SLS1* in the *kpc-1* 3′UTR abolished the ability of the *kpc-1* transgene to rescue tertiary dendrite self-avoidance defects in the *kpc-1(gk8)* null allele (Fig 5F). The *kpc-1* transgene with mutations that disrupt base-pairing in the stem region of SLS2 in the 3′UTR exhibited significantly more overlapped sibling tertiary dendrites (indicating losing self-avoidance functions; Fig 5C and 5F) whereas the *kpc-1* transgene with reciprocal mutations that preserve base-pairing in the stem region of SLS2 in the 3′UTR did not cause more overlapped sibling tertiary dendrites (indicating maintaining self-avoidance functions; Fig 5D and 5F). Using the MS2 tagging system, we further showed that deletion of SLS2 in the *kpc-1* 3′UTR significantly reduced the number of puncta and percentage of PVD neurons with MS2 signals on the tertiary dendrite (Fig 3G and 3H), suggesting that SLS2 is essential for *kpc-1* mRNA localization to the higher-order dendrites.

All the *kpc-1* transgenes tested here partially rescued secondary dendrite branching defects in the *kpc-1(gk8)* null allele, suggesting that the modifications we introduced to the transgenes, including the 3′UTR replacement, deletions, and mutations, did not result in degradation of the *kpc-1* transcripts (Fig 5E). Levels of the *kpc-1* RNA expressed from various transgenes in *kpc-1(gk8)* mutants were analyzed by RT-PCR. All *kpc-1* 3′UTR modifications were derived from the PF49H12.4::*kpc-1s*::*kpc-1* 3′UTR construct, where only the short-isoform *kpc-1s* RNA would be generated. We found that the *kpc-1* RNA expression levels are comparable among different *kpc-1* transgenes (S6 Fig), indicating that modifications we introduced to the *kpc-1* 3′UTR did not affect the *kpc-1* RNA stability. Furthermore, even though the level of the *kpc-1* RNA in wild-type animals (containing both long and short isoforms) is lower than the level of the *kpc-1* RNA in *kpc-1(gk8)* transgenic animals carrying high copy number of various *kpc-1* transgenes (containing only short isoform), dendrite self-avoidance is better in wild-type than transgenic animals (S6 Fig), suggesting the long isoform might be more efficient in promoting dendrite self-avoidance. Together, our studies identified an important secondary structural motif in the *kpc-1* 3′UTR required for mRNA localization to the higher-order dendrites (Fig 5G) and dendrite self-avoidance.

To validate the role of the *kpc-1* 3′UTR secondary structural motif SLS2 in PVD dendrite branching and self-avoidance, we applied the CRISPR-Cas9 engineering to generate mutations in the *kpc-1* 3′UTR. Due to the AT-rich sequence in the region surrounding the SLS2 site, only suboptimal sgRNAs with low target selectivity could be designed, preventing the generation of specific SLS2 mutations. A more distal but selective sgRNA was subsequently designed to generate a 284 base-pair deletion that includes the SLS2 site. Although transgene experiment using *kpc-1(gk8)* mutants carrying the *Ppvd*::*kpc-1*::*kpc-1* 3′UTR transgene with mutated SLS2 base pairing demonstrated an important role of SLS2 in dendrite self-avoidance, the CRISPR-Cas9 engineered *kpc-1(xr82) [SLS2 deleted]* mutant allele only showed a weak tertiary dendrite self-avoidance defect (S7A Fig). The mild phenotypic effects of the *kpc-1(xr82)* mutant allele were inadvertently caused by the creation of a new stem-loop structure that likely substitutes the SLS2’s function (S7C Fig). Although the *xr82 [SLS2 deleted]* allele alone did not cause a severe dendrite branching defect or severe self-avoidance defect, introducing the *xr84 [SLS2 deleted]* allele, as having the same SLS2 deletion as the *xr82* allele, into the *kpc-1(xr58)* background collectively caused significantly more dendrite branching defects compared to wild-type (S7B Fig). Our results supported that SLS2 in the *kpc-1* 3′UTR is required for dendritic transport of *kpc-1* mRNAs and PVD dendrite patterning.

### KPC-1 down-regulates DMA-1 receptors on PVD tertiary dendrites

**Fig 6:**
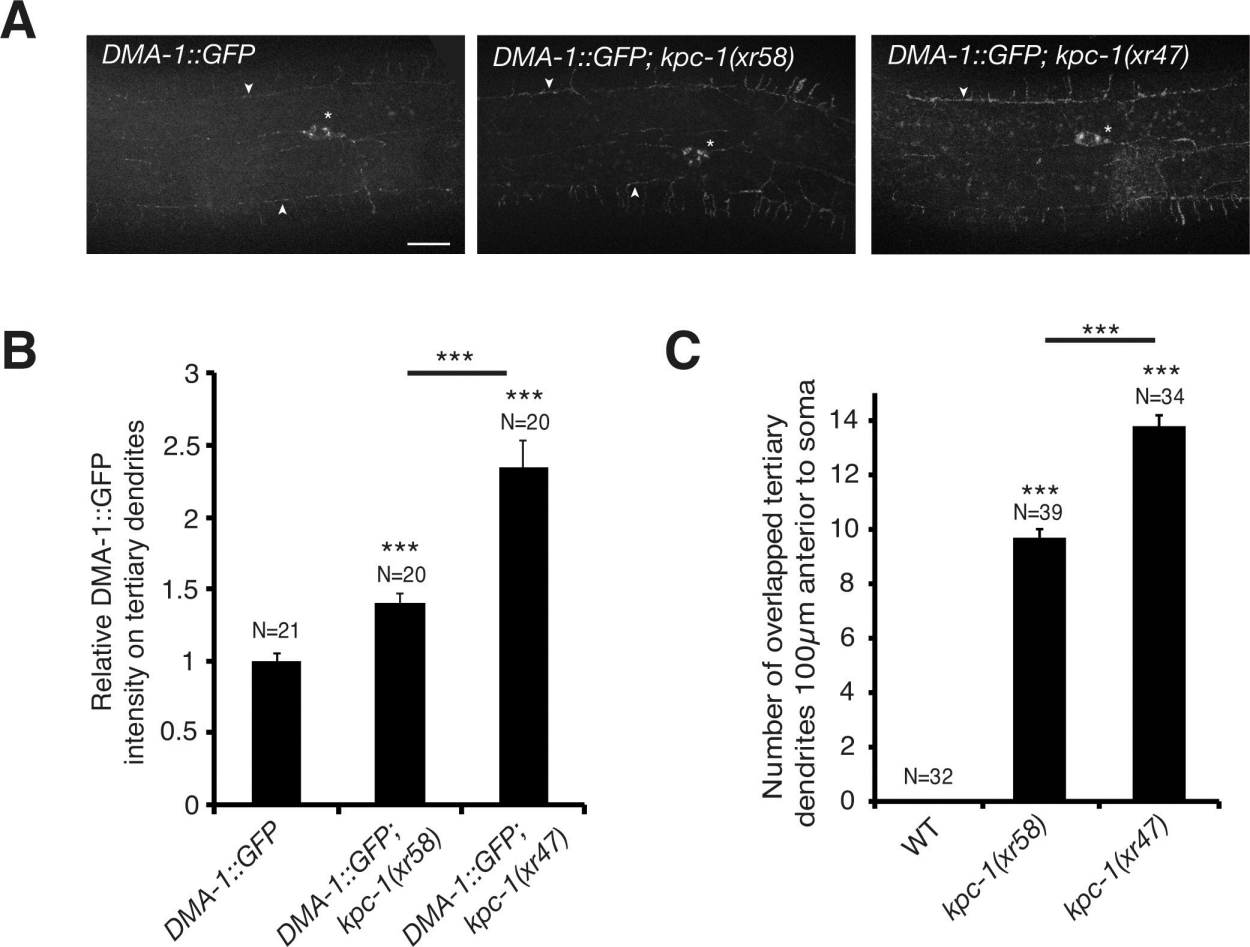
*kpc-1* alleles differentially affect DMA-1 protein levels on 3° dendrites and 3° dendrite self-avoidance. (A) Representative images showing *dma-1(wy1246)* [DMA-1::GFP] expression on PVD dendrites in wild-type, *kpc-1(xr58)*, and *kpc-1(xr47)* mutants. Asterisk indicates soma. Arrowheads indicate tertiary dendrites. Scale bars, 20 μm. (B) Average fluorescence intensity of the DMA-1::GFP on PVD tertiary dendrites in wild-type, *kpc-1(xr58)* mutants, and *kpc-1(xr47)* mutants. This reporter strain does not cause any defects in PVD dendrites [2]. ***p < 0.001 by one-way ANOVA with Tukey’s test. (C) Quantification of overlapped tertiary dendrites in wild-type versus *kpc-1* mutants. ***p < 0.001 by one-way ANOVA with Tukey’s test. The N number indicates the number of animals analyzed.

While the extent of tertiary dendrites outgrowth was significantly reduced in *sax-7*, *mnr-1*, *lect-2*, or *dma-1* mutants, it was significantly increased in *kpc-1(xr58)* mutants, as indicated by overlapping tertiary dendrites [15,16,18–20] (Fig 6C). These results suggested that *kpc-1* likely antagonizes the *sax-7/mnr-1/lect-2/dma-1* signaling pathway. The *kpc-1(gk8)* null allele seems to have a phenotype very similar to the *sax-7*, *mnr-1*, *lect-2*, and *dma-1* mutants [15,16,18–20] (Fig 2D). However, closer examination of the *kpc-1(gk8)* null mutant phenotype revealed that defective secondary dendrite extension to sublateral longitudinal stripes is caused by a failure of secondary dendrites branching out [3], a phenotype that is clearly different from the less secondary dendrite outgrowth in *sax-7*, *mnr-1*, *lect-2*, and *dma-1* mutants. Double mutant analyses between the *kpc-1(gk8)* null allele and *sax-7*, *mnr-1*, and *dma-1* mutations have been reported before, where mutations in *sax-7*, *mnr-1*, and *dma-1* suppressed the secondary dendrite branching defects in *kpc-1(gk8)* mutants [3], indicating that the *kpc-1* mutant effect depends on the *sax-7*/*mnr-1/lect-2*/*dma-1* signaling pathway. *dma-1*, like *kpc-1*, encodes a transmembrane protein and acts cell-autonomously in PVD neurons [3,18]. Thus, DMA-1 could be a potential KPC-1 target in regulating PVD tertiary dendrite self-avoidance. Supporting this possibility, endogenous DMA-1 protein levels on PVD tertiary dendrites were up-regulated in *kpc-1* mutants (Fig 6A and 6B), suggesting that KPC-1 normally down-regulates DMA-1 on PVD tertiary dendrites. In addition, the *kpc-1(xr47)* mutant allele, which showed a greater increase in the number of overlapped tertiary dendrites compared to the *kpc-1(xr58)* mutant allele (Fig 6C), caused a greater up-regulation of the DMA-1 protein level on PVD tertiary dendrites than the *kpc-1(xr58)* mutant allele (Fig 6A and 6B). A previous study reported a different phenotypic effect of *kpc-1* mutations in PVD neurons where *kpc-1* mutations caused excessive DMA-1 receptors on secondary dendrites, resulting in secondary dendrites trapped in the primary dendrite zone where a high level of the cognate ligand SAX-7/L1CAM is present [3].

### The *kpc-1-dma-1* regulatory circuit promotes dendrite self-avoidance

**Fig 7:**
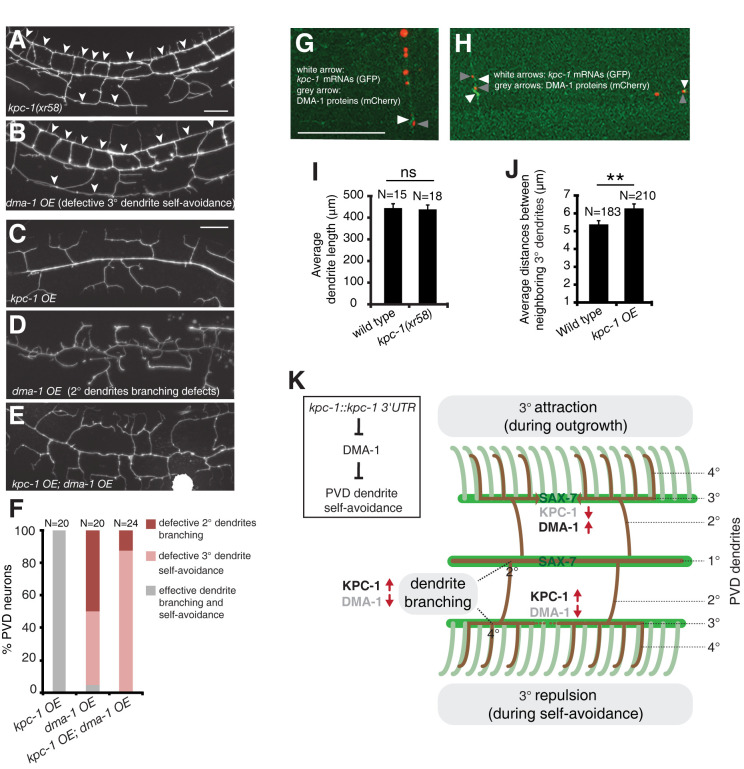
*dma-1* over-expression phenocopied the *kpc-1(xr58)* mutant allele. (A-B) Images of PVD dendrites in *kpc-1(xr58)* mutants and *dma-1* over-expression animals. Arrowheads point to contacts between neighboring tertiary dendrites. Scale bars, 20 μm. (C-E) Images of PVD dendrites in *kpc-1* over-expression animals, *dma-1* over-expression animals, and animals over-expressing both *kpc-1* and *dma-1*. Scale bars, 20 μm. (F) *kpc-1* over-expression suppressed secondary dendrite branching phenotypes in *dma-1* over-expression animals. (G-H) Images of PVD neurons showing dendritic signals for *kpc-1* transcripts and DMA-1 proteins. *kpc-1* transcripts containing *kpc-1* 3′UTR and MS2 binding sites were visualized by green fluorescent MS2 capsid proteins. DMA-1 proteins were visualized by tagged mCherry fluorescent proteins. White arrowheads point to dendritic signals for *kpc-1* transcripts, and grey arrowheads indicate dendritic signals for DMA-1 proteins. Scale bars, 15 μm. (I) Bar chart of average PVD dendrite length in wild-type and *kpc-1(xr58)* mutants. Dendritomy was performed at the early L4 stage when tertiary dendrites underwent self-avoidance. Newly grown PVD dendrite length was measured 24 hours after dendritomy. P = 0.83 by a Student’s *t*-test. Error bars indicate SEM. (J) Bar chart of average distances between neighboring tertiary dendrites in wild-type and *kpc-1* over-expression animals at the young adult stage. For each animal, average distances of the gaps between neighboring tertiary dendrites were measured per 150 μm anterior to the cell body. *kpc-1* over-expression animals differ from wild-type at **p<0.01 by a Student’s *t*-test. Error bars indicate SEM. (K) Model of the *kpc-1-dma-1* regulatory circuit in dendrite branching and self-avoidance. *kpc-1* is expressed at branching points and contact points between the neighboring 3° dendrites, which down-regulates DMA-1 receptors locally, leading to dendrite branching and retraction of contacted sibling dendrites. SAX-7 cues are distributed highly in the 1° and the 3° dendrite growth pathways. A simple genetic pathway illustrates the molecular control of dendrite self-avoidance.

To further strengthen the role of a *kpc-1-dma-1* regulatory circuit in promoting PVD dendrite self-avoidance, we tested whether *dma-1* over-expression (OE) mimics the *kpc-1* mutation in its phenotypic effect on PVD dendrites. *dma-1* OE displayed a tertiary dendrite self-avoidance defect similar to that exhibited by the *kpc-1(xr58)* mutant allele (Fig 7A and 7B). In addition, *dma-1* OE caused a secondary dendrite branching phenotype (Fig 7D), which was further enhanced by the *kpc-1(xr58)* mutation. In contrast, *kpc-1* OE suppressed the secondary dendrite branching phenotype in *dma-1* OE animals (Fig 7C–7F). While fewer *kpc-1* OE*; dma-1* OE animals displayed a secondary dendrite branching phenotype, most still exhibited a weak tertiary dendrite self-avoidance defect, which was milder than *dma-1* OE or *kpc-1(xr58)* alone. Thus, a weaker *kpc-1-dma-1* regulatory circuit (moderately elevated *dma-1*) causes tertiary dendrite self-avoidance defects whereas a much weaker *kpc-1-dma-1* regulatory circuit (strongly elevated *dma-1*) results in secondary dendrite branching defects. Indeed, the mild *kpc-1(xr58)* mutant allele mainly affects tertiary dendrite self-avoidance rather than secondary dendrite branching. Thus, our results support a model in which locally expressed KPC-1 down-regulates DMA-1 receptors on PVD dendrites to promote dendrite branching and self-avoidance (Fig 7K).

We have made different versions of KPC-1::dzGFP^myr^ and KPC-1::GFP fusion proteins, both wild-type and mutant forms that disrupted the KPC-1 self-cleavage site. However, we were unable to detect any fluorescence signal beyond the PVD cell body, suggesting that KPC-1 proteins have a very short half-life in PVD dendrites. Due to this technical limitation, we cannot directly determine whether KPC-1 proteins are synthesized locally in tertiary dendrites. Nor can we address whether the KPC-1 protein and its substrate DMA-1 protein colocate to higher-order dendrites. As an alternative approach, we superimposed the image of dendritic signals for *kpc-1* mRNAs with the image of dendritic signals for DMA-1 proteins. We found that they did not show an overlapping distribution pattern (Fig 7G and 7H). Instead, adjacent expression sites can be detected for *kpc-1* mRNAs and DMA-1 proteins (Fig 7G and 7H). While these results are consistent with the down-regulation of DMA-1 by KPC-1 at specific sites, without seeing colocalization first followed by a subsequent loss of colocalization, this statement may be premature.

The tertiary dendrite self-avoidance defect could result from either excessive dendrite growth ability that overcomes a self-avoidance mechanism or insufficient self-avoidance. To distinguish between these two possibilities, we performed laser dendritomy in PVD neurons at the early L4 stage when tertiary dendrites underwent self-avoidance and assessed the dendrite growth ability 24h after surgery. Dendrites were cut at the primary dendrite 20 μm anterior to the cell body such that the entire anterior dendrite arbor was disconnected from the cell body after surgery. We found that PVD dendrite growth ability was not significantly enhanced in *kpc-1(xr58)* mutants compared to wild-type animals (Fig 7I). This result indicated that the dendrite self-avoidance defect in *kpc-1(xr58)* mutants cannot be attributed to excessive dendrite growth ability. In contrast, over-expression of *kpc-1* moderately, albeit significantly, increased the distance (gap) between neighboring tertiary dendrites (Fig 7C and 7J). Together, these results support that *kpc-1* promotes dendrite self-avoidance rather than inhibiting initial dendrite outgrowth.

### Defective PVD dendrite arborization causes reduced male mating efficiency

**Fig 8:**
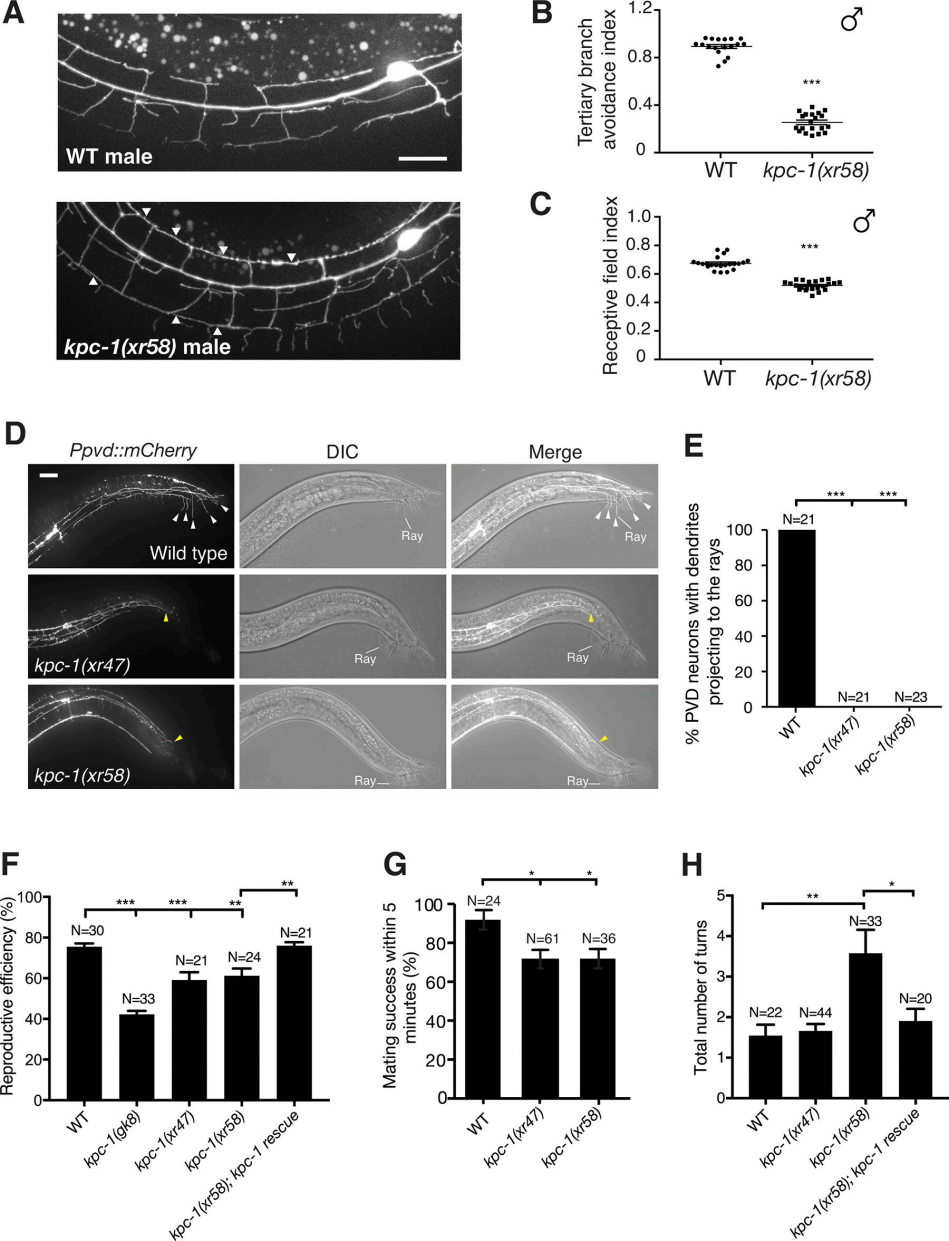
*kpc-1* mutant males exhibit mating defects. (A) Images of PVD dendrites in wild-type and *kpc-1(xr58)* mutant males. Arrowheads point to contacts between neighboring tertiary dendrites. Scale bars, 20 μm. Error bars, SEM. (B)Tertiary branch avoidance index of wild-type and *kpc-1(xr58)* mutant males. ***p < 0.001 by a Student’s *t*-test. Error bars, SEM. (C) Receptive field index of wild-type and *kpc-1(xr58)* mutant males. ***p < 0.001 by a Student’s *t*-test. (D) Images of PVD dendrites and the rays in wild-type and *kpc-1* mutant males. Scale bars, 20 μm. The location of the rays was indicated by the white line. White and yellow arrowheads point to the termini of PVD dendrite arbors in wild-type and *kpc-1* mutants, respectively. (E) Percentage of PVD neurons with dendrite projections to the rays. ***p < 0.001 by a two-proportion *Z*-test. (F) Reproductive efficiency of wild-type and *kpc-1(xr58)* mutant males. **p<0.01 and ***p < 0.001 by one-way ANOVA with Tukey’s test. Error bars, SEM. (G) Percentage of males that successfully transferred sperm within 5 min of first tail contact with a hermaphrodite. *p < 0.05 by one-way ANOVA with Tukey’s test. Error bars, SEM. (H) The number of turns the males made around hermaphrodites’ heads and tails before locating the vulva. *p<0.05 and **p < 0.01 by one-way ANOVA with Tukey’s test. Error bars, SEM.

The mechanical sensation is known to regulate animal locomotion and posture during male mating behavior [40–42]. PVD neurons are known mechanosensors but whether they are involved in male mating behavior is not known. Mature PVD dendrite arbors display a sensory network that innervates the skin area outside of the head region. We characterized PVD dendrites in *kpc-1(xr58)* mutant males and found that *kpc-1(xr58)* mutant males also displayed dendrite self-avoidance defects and reduced sensory coverage of skin by PVD dendrite arbors (Fig 8A–8C). In wild-type males, PVD dendrites always project posteriorly to the rays, composed of various sensory organs required for male mating. In contrast, in *kpc-1* mutant males, PVD dendrites fail to project to the rays (Fig 8D and 8E). Since the rays in male tails are known to regulate the contact of hermaphrodites and turning response in male mating behaviors [43], these results suggest *kpc-1* mutations could affect male mating behaviors. Consistent with having this neuronal connectivity defect, we found that the *kpc-1* mutant males exhibited significantly reduced reproductive efficiencies (Fig 8F), suggesting that PVD neurons are involved in the male courtship process. The male mating process begins with males responding to the hermaphrodite contact, turning around the hermaphrodite’s head and tail until the vulva is located. Both *kpc-1(xr47)* and *kpc-1(xr58)* mutant males showed decreased mating success within five minutes (Fig 8G). Moreover, *kpc-1(xr58)* mutant males are required to make more turns to mate with hermaphrodites successfully (Fig 8H), indicating a specific defect in turning response during mating. While the *kpc-1(xr47)* mutant showed reduced reproductive efficiency and mating success (Fig 8F and 8G), no increased number of turns before mating (Fig 8H) suggests that other stages of the mating sequence were affected. The defects in reproductive efficiency and the turning process in *kpc-1(xr58)* mutants can be rescued by the *kpc-1* transgene (Fig 8F and 8H). In summary, *kpc-1* patterns PVD neurons to coordinate diverse behavioral motifs in reproductive behavior. These results suggest that male mating behavior requires mechanosensory inputs from PVD neurons to generate proper mating motor patterns.

## Discussion

Our results support a model where locally synthesized KPC-1 at branching points and contact points between sibling dendrites down-regulates DMA-1 receptors to promote dendrite branching and self-avoidance (Fig 7K). A recent report showed that KPC-1 targeted DMA-1 to endosomes for degradation in PVD neurons [3]. It is likely that a similar mechanism is utilized in a site-specific manner for secondary dendrite branching [3] and tertiary dendrite self-avoidance. KPC-1 encodes a proprotein convertase subtilisin/kexin (PCSK). At a functional level, there are two types of mammalian PCSKs. PCSK3/furin activates substrates by cleavage of precursor proteins, such as TGF-β, whereas PCSK9 inactivates substrates by induced degradation of proteins, such as LDL receptors [44–47]. Our results indicate that KPC-1 is more closely related to PCSK9 at a functional level in its inactivation of DMA-1. It has been shown that PCSK9 is expressed in the vertebrate CNS, including the cerebellum, and is important for CNS development [48]. However, it remains to be seen whether PCSK9 plays a similar role to KPC-1 in dendrite branching and self-avoidance by down-regulating dendrite receptors.

It is necessary that the level of DMA-1 receptors on tertiary dendrites is tightly controlled during dendrite outgrowth and self-avoidance (Fig 7K). For example, the DMA-1 receptor level should be high on tertiary dendrites during dendrite outgrowth. However, the DMA-1 receptor level must be down-regulated upon contact between sibling tertiary dendrites, resulting in dendrite retraction (self-avoidance). An important question that remains to be addressed in the future is how the KPC-1 activity is dynamically controlled to regulate corresponding DMA-1 levels. There are two possible regulatory mechanisms that could be responsible: expression versus activation of KPC-1 at the contact points between sibling dendrites. Our observation of the enrichment of *kpc-1* transcripts at the contact points appears to support the former case (Fig 3E). However, it is unknown how *kpc-1* transcripts are targeted to contact points and whether contact-triggered RNA transport is responsible.

Although our results demonstrate the important role of the *kpc-1* 3′UTR in promoting *kpc-1* mRNA transport to the PVD higher-order dendrites, questions remain on how the 3′UTR elements interact with the RNA transport system. It was recently reported that 12 conserved RNA-binding protein (RBP)-encoding genes are involved in PVD dendrite development in *C*. *elegans* [49]. Among them, loss of function in *cgh-1* and *cpb-3* resulted in an increase in the number of tertiary dendrite branches. The phenotype of an increased number of tertiary dendrite branches is also shared by *kpc-1(xr58)* mutants (Fig 1A). Like *kpc-1*, *cgh-1* and *cpb-3* act cell-autonomously in PVD neurons to control dendrite development. Also, like *kpc-1* transcripts, CGH-1 and CPB-3 proteins are enriched in PVD dendrites, raising the possibility that CGH-1 and CPB-3 may regulate the *kpc-1* function in dendrites by binding to the 3′UTR of *kpc-1* mRNAs, a possibility awaiting to be explored.

A Schizophrenia-associated genetic variant in the 3′UTR of the human furin gene, a *kpc-1* homologous gene, was recently shown to result in downregulation of furin expression by acquiring a miR-338-3p binding site, leading to reduced BDNF production. It remains to be seen whether this human furin 3′UTR genetic variant disrupts transport and local translation of the furin mRNA in dendrites and whether it affects dendrite patterning in Schizophrenia-causing neurons [50,51].

Even though mechanical sensation is known to regulate animal locomotion and posture during male mating behavior[43], it is unknown whether PVD neurons, best known for their roles in harsh mechanical sensations of body stimulation, modulate male mating behavior. In this report, we showed that PVD dendrites in *kpc-1* mutant males fail to project to the rays in tails (Fig 8D and 8E), where various sensory organs important for the regulation of the hermaphrodite contact and turning response in male mating behaviors are located, suggesting a regulatory role of PVD neurons in the male mating process. Indeed, consistent with this neuronal connectivity defect, we found that the *kpc-1* mutant males exhibited significantly reduced reproductive efficiencies as well as defects in specific behavioral motifs in reproductive behavior (Fig 8F–8H).

## Materials and methods

### Strains and plasmids

*C*. *elegans* strains were cultured using standard methods [52]. All strains were grown at 20°C. Standard protocol was used for the plasmid constructions. Strains and plasmids are listed in S1 and S2 Tables.

### Isolation of *kpc-1* alleles from the genetic screen

A forward genetic screen was conducted as previously described. Wild-type worms with *xrIs37* marker were treated with EMS [3]. F1 progenies were transferred to single plates, and F2 progenies were screened for profoundly affected dendrite self-avoidance defect. Using a Zeiss fluorescence dissecting microscope. The *kpc-1(xr47)*, *kpc-1(xr58)*, and *kpc-1(xr60)* mutations were identified from a screen of 9,500 genomes.

### Transgenic animals

Germline transformation of *C*. *elegans* was performed using standard techniques [53]. For example, the *Pkpc-1(5K)*::*kpc-1* transgene was injected at 10 ng/ml along with the coinjection marker *ofm-1*::*rfp* at 50 ng/μl. Transgenic lines were maintained by following the *ofm-1*::*rfp* fluorescence.

### Tertiary branch avoidance index (TBAI)

TBAI was calculated by dividing the number of visible gaps between tertiary branches by the number of menorahs. A menorah-like structure is composed of a collection of 2° (“stem”), 3° (“base”) and 4° (“candles”) branches. In the case where discrete menorahs cannot be easily differentiated due to self-avoidance defects, we counted each menorah as a single 2° (“stem”) branch that extended 3° (“base”) branches.

### Determination of receptive field index

The receptive field index was defined by the ratio of the skin area innervated by the PVD anterior dendrite arbors normalized to the skin area of the animal body, measured from the PVD soma to the end of the mouth (anterior most point in the animal).

### Measurement of menorah coverage zone and trapped 2° dendrite zone

We measured the “menorah coverage zone” as an area not interrupted by trapped 2° dendrites rather than an area of the individual dendrites. A schematic depicting menorah coverage zones and trapped 2° dendrite zones was shown in S1G Fig. The menorah coverage zone was measured by the average length of menorah coverage on the ventral and dorsal side over 200 μm of 1° dendrite. The trapped 2° dendrite zone was measured by the average length of trapped 2° dendrites on the ventral and dorsal side over 200 μm of 1° dendrite.

### Scatter plots of quaternary dendrite termini

To map out quaternary dendrite termini, we used a set of customized MatLab scripts (MatLAB, Mathworks, Natick, MA) to effectively unroll the worm’s cylindrical surface, quantifying anterior-posterior distances using the coordinate parallel to the body centerline and dorsal-ventral distances along the cylindrical surface. In order to consolidate data from different worms, all scatter plots are scaled to each worm’s circumference. In each scatter plot, the top line indicates the dorsal nerve cord, the bottom line indicates the ventral nerve cord, and the wild-type morphology of the PVD primary dendrite and axon is drawn in green. The distance between the top and bottom lines corresponds to 1/2 of total worm circumference, and the horizontal axis shows a portion of body length equivalent to approximately 3 circumferences.

### Molecular cloning

All constructs generated for this study are summarized in S2 Table. Sequence variants of the *kpc-1* 3′ UTR were verified by sequencing. Oligonucleotides used are listed in S3 Table.

### CRISPR-Cas-9 genome editing

To generate deletion of the SLS2 region in the *kpc-1* 3′ UTR, we used the Co-CRISPR *dpy-10(cn64)* as a screening method [54]. Worms were injected with Cas9_sgRNA plasmid and single-stranded oligonucleotide donor (IDT). Both *xr82* and *xr84* SLS2 deletion alleles were confirmed by sequencing. Oligonucleotides used are listed in S3 Table.

### Laser dendritomy

For the femtosecond laser surgery, we used a cavity-dumped Ti:sapphire laser oscillator [55,56] to generate laser pulses ~100 fs in duration and 200 kHz in repetition rate. The laser pulses were tightly-focused onto targeted primary dendrites using a Nikon 100x, 1.4 NA oil-immersion objective. The vaporization threshold corresponds to pulse energies of 5–15 nJ. Successful laser dendritomy was confirmed by visualizing the targeted area immediately after surgery. Dendrites were cut at the primary branch 50 μm anterior to the cell body in the early L4 stage when tertiary dendrites undergo self-avoidance. 1°, 2°, 3°, and 4° dendrites in the anterior dendrite arbor were disconnected from the cell body after primary dendritomy. Newly regenerated dendrites can be easily differentiated from the old dendrites 24 hr after surgery due to difference in the fluorescence intensity of the *Pser-2*::*GFP* dendrite marker. All the newly regenerated dendrite branches were quantified using ImageJ.

### Imaging and quantifying dendrite arbors

The morphology of neuronal cell bodies and dendrites was based on high-magnification Z-stacks using a Zeiss 60x, 1.4 NA oil-immersion objective. For laser dendritomy, we mounted individual animals on 2% agar pads and anaesthetized them with 3 mM sodium azide, the lowest possible concentration to keep adult animals immobilized. Laser dendritomy was performed and worms were recovered within 10 minutes of sodium azide treatment. Recovered worms were placed on fresh plates with bacterial foods and imaged 24 hours after dendritomy using a Hamamatsu ORCA AG camera. For imaging and quantifying uninjured dendrite arbors, young adult animals were mounted on 2% agar pads and anaesthetized with 20 mM sodium azide.

The dendrite length of regenerating neurons was quantified 24 hours after surgery. Dendrite lengths were calculated as the actual contour length between the injury site and dendrite termini measured along the cylindrical surface of each worm, by tracing dendrites through a 3-dimensional image stack. P values for the length measurements were calculated using a student’s t-Test.

### Fluorescence microscopy

Worms were mounted on 2% agarose pad and anaesthetized with 20 mM sodium azide solution. Images were acquired using a 40x, 1.4 NA objective on a Zeiss Axio Imager M2 microscope with a Hamamatsu ORCA-Flash4.0 LT+ camera. Images were processed by z-stack projections. Image analyses of dendrite branching and self-avoidance phenotypes, mRNA localization, DMA-1 protein level on dendrites, dendrite growth ability, and Fluorescence recovery after photoconversion were performed at young adult stage unless otherwise specified.

### Fluorescence recovery after photoconversion

Worms were mounted on 2% agarose pad and anaesthetized with 7.5 mM tetramisole.

Photoconversion of Kaede was performed under a 405 nm laser on the region of interest (ROI). ROI (20μm x 10μm) was consistently chosen near the distal end of the anterior PVD primary dendrite. Fluorescence quantification of ROI intensity was measured every 5 seconds over 6 minutes following photoconversion (Zeiss Imaging Browser). At each time point, the percentage of green fluorescence signal recovery was determined by (F_n_-F_0_)/(F_p_-F_0_), where F_n_ = fluorescence intensity n seconds after photoconversion; F_0_ = fluorescence intensity immediately after photoconversion; F_p_ = fluorescence intensity right before photoconversion. All images were taken in live animals using a 40x, 1.3 NA objective, and 488 nm and 561 nm laser on a Zeiss LSM 880 confocal microscope.

### Male mating behavior assays

Male mating behavioral assays were conducted using standard methods [43]. Reproductive efficiency assays were conducted by placing assayed males and hermaphrodites carrying the *unc-36(e251)* recessive marker. Reproductive efficiency results were calculated by the number of cross-progeny over the total number of progenies. Assays for determining the number of hermaphrodites contacted before mating initiation and the total number of turns around hermaphrodites during mating were conducted according to the methods previously described [42]. Young adult *unc-36(e251)* hermaphrodites were used for all male mating behavior assays.

### Statistics

Average data of dendrite number, dendrite length, TBAI, receptive field index, and reporter expression intensity are presented as means ± SEM. Data of % PVD neurons with secondary dendrite branching defects, defective dendrite self-avoidance, and dendritic signals are presented as proportions ± SEP. Statistical analyses were carried out by Student’s *t*-tests, two-proportion *Z*-tests, one-way ANOVA with Tukey’s or Dunnett’s tests, or two-way ANOVA using GraphPad Prism 7.0 or the Primer of Biostatistics software.

## Supporting information

S1 Fig Characterization of *kpc-1* mutant alleles. A-E. Representative images of PVD dendrites in wild-type, *kpc-1(xr58)*, *kpc-1 (xr47)*, *kpc-1(xr60)*, and *kpc-1(gk8)* mutants. Scale bar, 20 μm. F. Schematic of KPC-1 protein motifs showing locations of *xr47*, *xr58*, *xr60*, and *gk8* mutations. G. Schematic of *kpc-1(xr60)* mutant phenotype with menorah coverage zone are labeled in black and trapped 2° dendrite zone labeled in red. H. Table of menorah coverage zone in the *kpc-1* mutants. The length of the menorah coverage zone is the average of the ventral and dorsal sides of PVD neurons. I. Table of trapped 2° dendrite zone in the *kpc-1* mutants. The length of the trapped 2° dendrite zone is the average of the ventral and dorsal sides of PVD neurons.
(EPS)

S2 Fig Time-lapse imaging of PVD dendritic arborization in wild-type and *kpc-1(xr58)* mutants. Time series images of tertiary dendritic arborization in wild type and *kpc-1(xr58)* mutants are shown. PVD neurons were imaged at 2.5–30 min intervals for 2.5 hours at the late L3 stage using confocal fluorescence microscopy. Arrowheads point to the dynamic contact points. Scale bar, 20 μm.
(EPS)

S3 Fig Measurement of sensory coverage of skin by PVD dendrite arbors. A, B Superimposed images of PVD dendrite arbors and animal bodies in wild-type (A) and *kpc-1(xr58)* mutants (B). C Receptive field index of the wild-type and *kpc-1(xr58)* mutants. ***p < 0.001 by a Student’s *t*-test. Error bars, SEM. D Summary of *kpc-1(xr58)* mutant phenotype.
(EPS)

S4 Fig Alignment of the 3′UTR of 4 *kpc-1* and the 3′UTR of 4 *unc-54* nematode homologs. A MAFFT alignment of 4 *kpc-1* 3′UTR sequences shown here corresponds to the following subsequences: *C*. *elegans*, nt 1–357; *C*. *remanei*, nt 1–366; *C*. *briggsae*, nt 1–340; *C*. *japonica*, nt 1–452. Stem-loop structure 1 (SLS1) and 2 (SLS2) regions are indicated. B MAFFT alignment of 4 *unc-54* 3′UTR sequences shown here corresponds to the following subsequences: *C*. *elegans*, nt 1–468; *C*. *remanei*, nt 1–390; *C*. *briggsae*, nt 1–280; *C*. *japonica*, nt 1–295. No conserved stem-loop structure was identified.
(EPS)

S5 Fig Assessment of the ability of different transgenes to rescue dendrite self-avoidance defects in *kpc-1(xr58)* mutants. Quantification of overlapped tertiary dendrites 200 μm anterior to soma in wild-type and *kpc-1(xr58)* mutants carrying various versions of transgenes. ***p<0.001 by one-way ANOVA with Dunnett’s test. The N number indicates the number of animals analyzed.
(EPS)

S6 Fig The *kpc-1* mRNA expression levels are comparable among different *kpc-1* transgenes. A RT-PCR was performed to determine the endogenous *kpc-1* mRNA levels in wild-type and *kpc-1(gk8)* mutants using an oligo(dT) RT primer and a pair of specific PCR primers to amplify a *kpc-1* fragment that is uncovered in the *kpc-1(gk8)* deletion allele. B The transgenic *kpc-1* mRNA levels in *kpc-1(gk8)* mutants expressing different *kpc-1* transgenes measured by RT-PCR. Actin (*arx-1*) was used as an internal control. C Schematic of the *kpc-1* cDNA. The primers used for amplifying *kpc-1* fragment from the transgenic *kpc-1* mRNA are labeled.
(EPS)

S7 Fig CRISPR engineering of *kpc-1* 3′ UTR and its effects on PVD dendrite arborization. A Percentage of PVD neurons with defective dendrite self-avoidance. *kpc-1(xr82) [SLS2 deleted]* was created by the CRISPR engineering. Defective dendrite self-avoidance was defined as more than one tertiary dendrite contact point for each PVD neuron. B Secondary dendrite branching defects measured by the trapped area. *kpc-1(xr58 + xr84[SLS2 deleted])* was generated in the *xr58* background in a separate CRISPR event from *xr82*, and both *xr82* and *xr84* alleles contain the same deletion that uncovers SLS2. The defect of secondary dendrite branching was qualitatively determined to be no (0), mild (1), moderate (2), and severe (3) defects. **p<0.01 and ***p<0.001 by one-way ANOVA with Dunnett’s test. C Schematic of the new SLS generated following the deletion in *xr82* and *xr84* alleles.
(EPS)

S1 Table A strain list. (XLSX)

S2 Table A Plasmid list. (XLSX)

S3 Table Oligonucleotides used in this study. Sequence information was searchable in the Wormbase.
(XLSX)
